# Real-time administration of indocyanine green in combination with computer vision and artificial intelligence for the identification and delineation of colorectal liver metastases

**DOI:** 10.1016/j.sopen.2023.03.004

**Published:** 2023-03-02

**Authors:** Niall P. Hardy, Jonathan P. Epperlein, Jeffrey Dalli, William Robertson, Richard Liddy, John J. Aird, Niall Mulligan, Peter M. Neary, Gerard P. McEntee, John B. Conneely, Ronan A. Cahill

**Affiliations:** a UCD Centre for Precision Surgery, School of Medicine, UCD, Dublin, Ireland; b IBM Research Europe, Dublin, Ireland; c Department of Histopathology, Mater Misericordiae University Hospital, Dublin, Ireland; d Department of General and Colorectal Surgery, University Hospital Waterford, University College Cork, Ireland; e Department of Hepatobiliary, Foregut and Bariatric Surgery, Mater Misericordiae University Hospital, Dublin, Ireland; f Department of General and Colorectal Surgery, Mater Misericordiae University Hospital, Dublin, Ireland

**Keywords:** Fluorescence guided surgery, Fluorescence quantification, Artificial intelligence, Colorectal liver metastases, Liver surgery, Indocyanine green

## Abstract

**Introduction:**

Fluorescence guided surgery for the identification of colorectal liver metastases (CRLM) can be better with low specificity and antecedent dosing impracticalities limiting indocyanine green (ICG) usefulness currently. We investigated the application of artificial intelligence methods (AIM) to demonstrate and characterise CLRMs based on dynamic signalling immediately following intraoperative ICG administration.

**Methods:**

Twenty-five patients with liver surface lesions (24 CRLM and 1 benign cyst) undergoing open/laparoscopic/robotic procedures were studied. ICG (0.05 mg/kg) was administered with near-infrared recording of fluorescence perfusion. User-selected region-of-interest (ROI) perfusion profiles were generated, milestones relating to ICG inflow/outflow extracted and used to train a machine learning (ML) classifier. 2D heatmaps were constructed in a subset using AIM to depict whole screen imaging based on dynamic tissue-ICG interaction. Fluorescence appearances were also assessed microscopically (using H&E and fresh-frozen preparations) to provide tissue-level explainability of such methods.

**Results:**

The ML algorithm correctly classified 97.2 % of CRLM ROIs (n = 132) and all benign lesion ROIs (n = 6) within 90-s of ICG administration following initial mathematical curve analysis identifying ICG inflow/outflow differentials between healthy liver and CRLMs. Time-fluorescence plots extracted for each pixel in 10 lesions enabled creation of 2D characterising heatmaps using flow parameters and through unsupervised ML. Microscopy confirmed statistically less CLRM fluorescence vs adjacent liver (mean ± std deviation signal/area 2.46 ± 9.56 vs 507.43 ± 160.82 respectively p < 0.001) with H&E diminishing ICG signal (n = 4).

**Conclusion:**

ML accurately identifies CRLMs from surrounding liver tissue enabling representative 2D mapping of such lesions from their fluorescence perfusion patterns using AIM. This may assist in reducing positive margin rates at metastatectomy and in identifying unexpected/occult malignancies.

## Introduction

Colorectal cancer is the third most common adult malignancy and up to one third of patients develop colorectal liver metastases (CRLM) during the course of their disease [1]. Surgery remains the only curative treatment option with advances in neoadjuvant therapies, understanding of disease biology and improved patient selection permitting more patients with metastases to be treated with curative intent [[2], [3], [4]]. Minimally invasive surgery (MIS) for CRLMs however has been slow in its adoption outside of specialist centres despite accruing evidence for its benefit [[5], [6], [7]]. Purported reasons for this include difficulty of access and localisation especially for posterior lesions (where often therefore requiring formal anatomical resections even for small lesions), restricted ergonomics (especially in the case of standard laparoscopy), the lack of tactile feedback and the risk of uncontrollable haemorrhage [8]. Improved intraoperative visualization could help offset these difficulties.

Indocyanine green (ICG), the protein-bound fluorophore, was serendipitously discovered to localise liver lesions in 2009 by Ishizawa et al., as part of a liver function study [9]. Since then, it has been increasingly tried as a means to localise liver lesions, especially during MIS using widely available near infrared (NIR) imaging systems. All studies investigating its use to date however have utilized antecedent dosing strategies with ICG administration up to 14 days prior to surgery. Such an approach relies on complete washout of ICG from surrounding healthy tissue and retention in, or around, lesions of interest. Reported tumour detection rates with such methods have varied widely from 43 to 100 % with false positive rates between 0 and 31.3 % and no consensus as of yet on dose amount or timing of administration [10].

Previous work by this group has shown the successful application of real-time ICG administration and artificial intelligence (AI) augmented interpretation of resulting dynamic perfusion profiles to characterise malignancies of the rectum based on the biophysical understanding of cancerous tissue, distinct from that of surrounding healthy rectal mucosa [[11], [12], [13]]. Here we apply this methodology to the liver, utilizing existing knowledge of ICG-liver interactions in combination with state-of -the-art ML methodologies to characterise and delineate CRLMs with reporting as per the DECIDE-AI guidelines for the early-stage evaluation of clinical support systems [14,15].

## Methods

Consenting patients undergoing diagnostic or therapeutic surgery for CRLMs at a high-volume liver surgery unit within a university teaching hospital were included in this prospective trial (NCT 04220242, Institutional Review Board (IRB) approval reference: 1/378/2092). Capsular and shallow subcapsular liver lesions were identified from staging scans in patients with colorectal cancer and were imaged in this study while undergoing either laparoscopic treatment of the primary or both open and minimally invasive metastatectomy (with subsequent confirmation of lesion nature using intraoperative ultrasound). All non-pregnant patients ≥ 18 years of age with clinical features suspicious of or diagnosed CRLM without known allergy to ICG or iodine were eligible for inclusion in the study.

Videos of ICG perfusion angiograms were created by injecting 0.05 mg/kg of ICG intravenously with the liver lesion alongside an area of healthy parenchyma for analytical comparison under direct, simultaneous observation with a commercially available NIR imaging system (three different systems were used namely Pinpoint, Stryker Corp, Kalamazoo, MI, USA, Elevision, Medtronic, Ireland and Firefly, Intuitive Surgical Inc., Sunnyvale, California, USA). Recordings were obtained for at least 90 s to ensure inflow, peak, and early egress of ICG was visualized in both healthy and unhealthy regions. The 0.05 mg/kg dose was chosen to reduce background fluorescence within the healthy liver parenchyma (which concentrates ICG) which was noted in preliminary work to cause signal saturation when higher doses were used [16].

As a first step, user selected ROI characterisation was evaluated. To do this, time-fluorescence curves were extracted from multiple user-selected tissue regions within the video recordings with each region annotated as “normal” or “abnormal”. Time series were created using a bespoke tracker-quantifier utilizing the white light video source for tracking and extracting the correlating fluorescence intensity from the synchronous NIR image at 30 frames per second on a standard laptop computer [17]. As the Firefly system alone does not facilitate synchronous white light and NIR video output, but does present a fixed image, the NIR green overlay video obtained was first converted to greyscale and fluorescence intensity extraction performed without concomitant white light tracking.

Prior to the application of AIM, manual data analysis was performed via the comparison of previously reported determinant fluorescence curve milestone ‘features’ (Time-to-Peak/Upslope/Time-Ratio/Downslope) with modifications to account for the liver as an ICG concentrating organ. After recording on Microsoft Excel worksheets, feature measurements were tested for normality using Shapiro-Wilk test and compared between groups (cancer vs healthy) using the Mann-Whitney *U* test. Statistical analysis was performed using SPSS Statistics V.27 (IBM, Armonk, NY, USA). Downslope readings were assessed from peak inflow values to 10, 20 and 60 s after the peak of inflow. Due to the absence of early timeframe peak in liver tissue, the equivalent CRLM peak was mapped to the healthy curve (or average peak time where more than one ROI was tracked) and slope calculated in both from that point.

The discriminatory ability (cancer vs healthy) of ROI data were subsequently tested using MATLAB (software version R2022b) to identify an optimised classifier by searching over all possible hyper-parameters for a large range of known classifiers to choose the best candidate based on accuracy. Training-testing was performed using 10-fold cross validation with all available ROIs with an 80:20 training:testing split.

Next, unsupervised 2D perfusion profile maps were created through video stabilization with post processing at 7 frames per second (permitting extraction of fluorescence intensity values for the entire image instead of user defined ROIs used in the ROI based classifier). The 10 most stable videos (minimal camera motion and respiration artefact) were chosen for such processing. Motion-camera and tissue deformation (camera/patient movement and respiration) movements were compensated by estimating the movement of pixels between frames and applying the estimated motion in reverse. For this, recognizable landmarks in each pair of frames were identified and matched and their relative motion computed, allowing interpolation of the motion of all other pixels using a thin-plate spline. Following such stabilization, pixel-by pixel time-fluorescence curves were extracted, and the data displayed as 2D images using piece-wise constant approximations of the profiles followed by unsupervised clustering as well as centre of mass (COM) and outflow slopes (displayed using a yellow-green-blue/viridis scale). Fluorescence curves with delayed COM (right shift on the x axis) were represented in yellow and those whose COM was earlier in the time series represented in blue. Outflow slopes were calculated between the timepoints “peak” and “peak +10 seconds” with red indicating a negative slope and blue positive on 2D recreation. The single benign liver lesion was also included as a demonstrator of 2D appearances in such an anomaly.

Pathological samples were taken from four patients for microscopic correlation with macroscopic appearances observed intraprocedurally using a Nikon Eclipse Ti2 Inverted Research Microscope and a LI-COR Odyssey DLx Near-Infrared *Fluorescence* Imaging System. Representative samples from resected specimens were taken incorporating a section of CRLM as well as a margin of surrounding healthy tissue. Specimens were mounted in OCT, flash frozen using Lamb's freezing aerosol and cut using a cryotome to 5 micrometre thick levels. Fluorescence intensities were compared between healthy liver and CRLM segments. Specimens were analysed in fresh frozen form with and without standard haematoxylin and eosin (H&E) staining with cover slipping to assess for the impact of such preparation on the fluorescence signal.

## Results

**Fig. 1:**
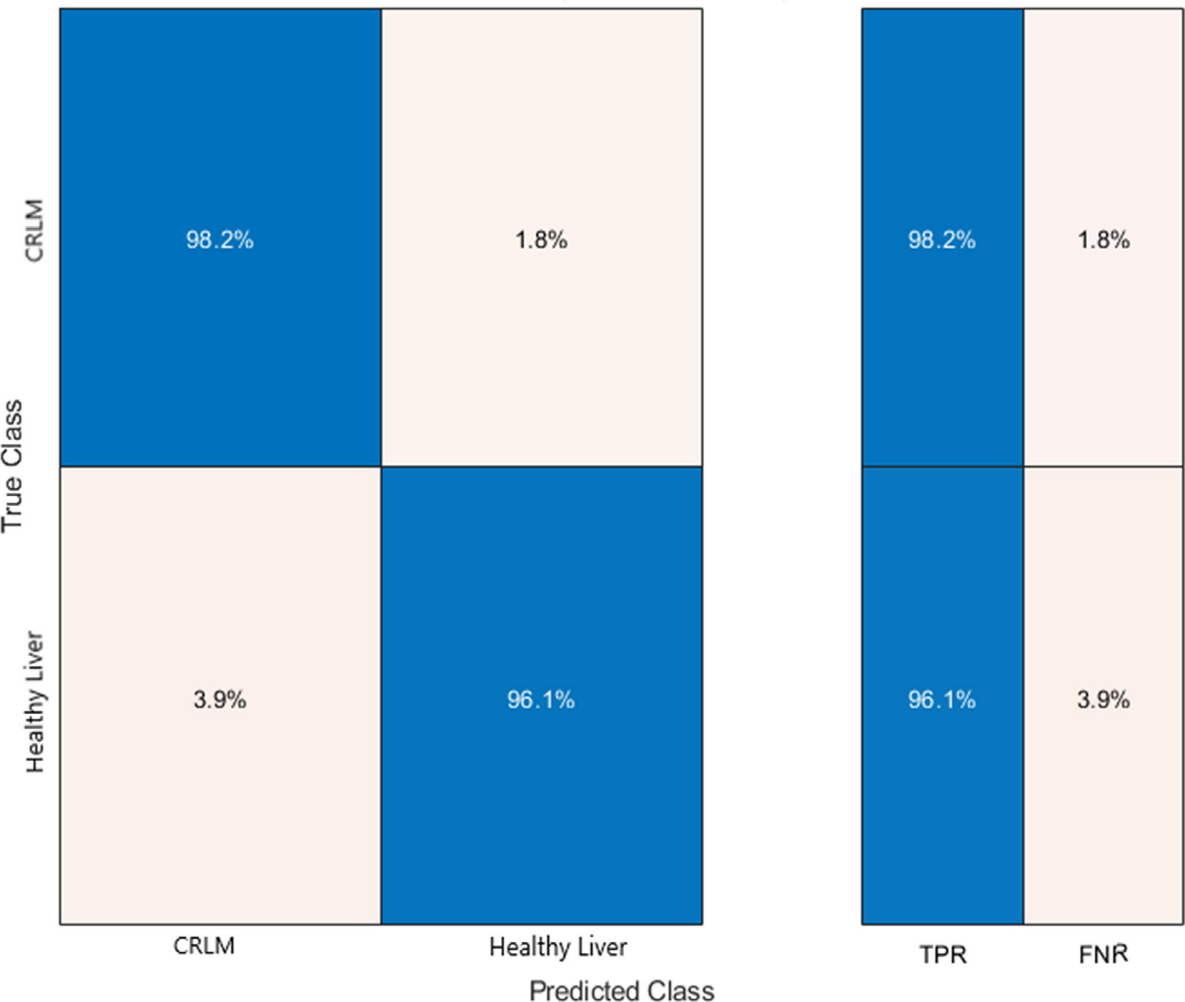
Results of optimised trees classifier following training and testing (with 10-fold cross validation) on 25 liver lesions (presented as a confusion matrix) along with true positive rates (TPR) and false negative rates (FNR). (CRLM = colorectal liver metastases.)

Video recordings of 25 consenting patients with liver lesions were analysed, 24 patients had CRLMs and one had a benign liver cyst (see Table 1 for patient demographics). Twenty-one patients were imaged using the PinPoint NIR imaging system by Novodaq/Stryker (20 CRLMs and 1 benign cyst), two with Medtronic's Elevision and two using the FireFly by Intuitive Surgical. Time-fluorescence profiles of 90 second duration were created for both lesion and healthy liver ROIs in all videos resulting in 132 CRLM and 6 benign cyst ROIs comprising 372,324 data points. Mathematical assessment of these extracted curves yielded significance in numerous time-series milestones with a concentration of significance around outflow parameters (see Table 1). An “optimisable tree” classifier demonstrated an average accuracy of 97.2 % for classifying CRLM ROIs and correctly identified all benign liver cyst ROIs with a positive predictive value of 92.3 % (see Fig. 1 for confusion matrix).

**Table 1:** Patient demographics and tabulated results of graph parameters obtained using the fluorescence intensity tracker. g.u. = greyscale units.

Malignant liver lesion characteristics	N = 24
Male:female	21:3
Age in years (mean ± std dev)	61 ± 11.77
NIR system used (PinPoint:Firefly:Elevision)	20:2:2


Liver variables	Mean values ± std dev		p values by Mann Whitney-U testing (significance <0.05)
	Colorectal liver metastases (n = 24)	Healthy liver tissue (n = 24)	
Tmax (time to peak intensity) (s)	23.931 ± 13.370	82.616 ± 12.888	<0.001*
Fmax (peak intensity) (g.u.)	123.594 ± 51.253	195.322 ± 57.883	<0.001*
End intensity (g.u.)	102.861 ± 48.899	188.988 ± 57.948	<0.001*
Upslope	6.951 ± 4.458	2.660 ± 1.446	<0.001*
Time ratio (T_1/2_/T _max_)	0.341 ± 0.208	0.127 ± 0.076	<0.001*
Intensity change: peak to peak+10 s (g.u.)	−24.471 ± 13.700	36.726 ± 30.341	<0.001*
Slope 10 s from peak	−2.447 ± 1.370	3.672 ± 3.034	<0.001*
Slope 20 s from peak	−1.190 ± 0.727	0.831 ± 0.837	<0.001*
Slope 60 s from peak	−0.283 ± 0.326	0.673 ± 0.488	<0.001*
Kurtosis	2.053 ± 2.037	1.366 ± 2.164	0.022*
Skew	−1.399 ± 0.966	−1.495 ± 0.589	0.572
Centre of mass	49.536 ± 6.504	53.995 ± 3.262	<0.001*

Significant results (p <0.05) marked with *.

**Fig. 2:**
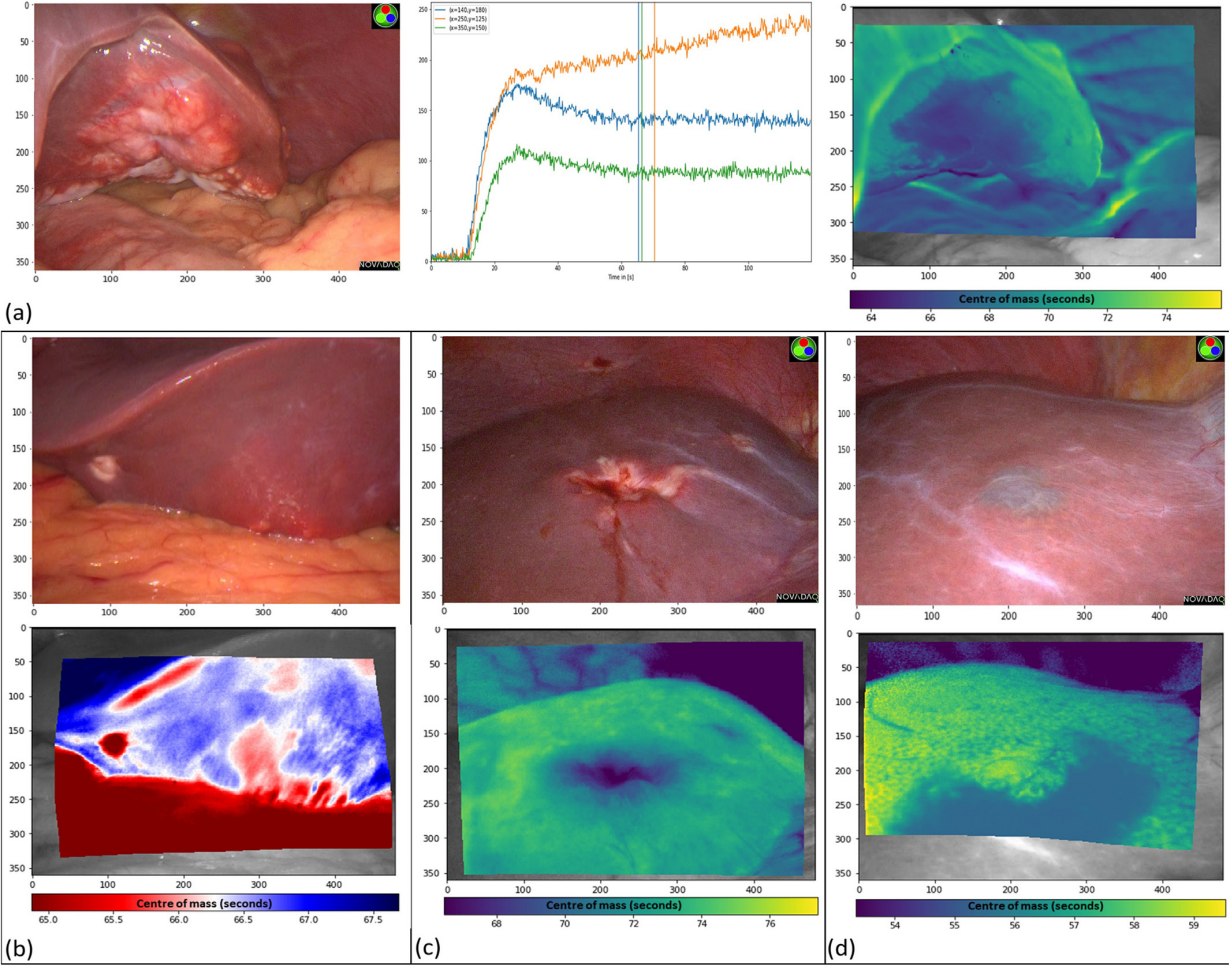
Images showing malignant liver lesions in white light as well as 2D images based on dynamic time-fluorescence profiles. Outer grey border around images represents regions lost during tracking. X, Y co-ordinates are constant across all images allowing image to image comparison. (a) White light view of colorectal liver metastasis with graph showing three example time-fluorescence curves, with X, Y co-ordinates, along with the centre of mass for each curve (vertical lines). Resulting heatmap generated utilizing all trackable pixels also shown. (b) White light view of colorectal liver metastasis with second “hidden” lesion demonstrated on heatmapping. Lesion was not evident on initial visual inspection but subsequently seen on intra-operative ultrasound. (c) Malignant liver lesion in white light with centre of mass heatmapping demonstrating lesion delineation. (d) Benign liver lesion (cyst) in white light demonstrating lack of lesion margination compared to surrounding healthy liver.

**Fig. 3:**
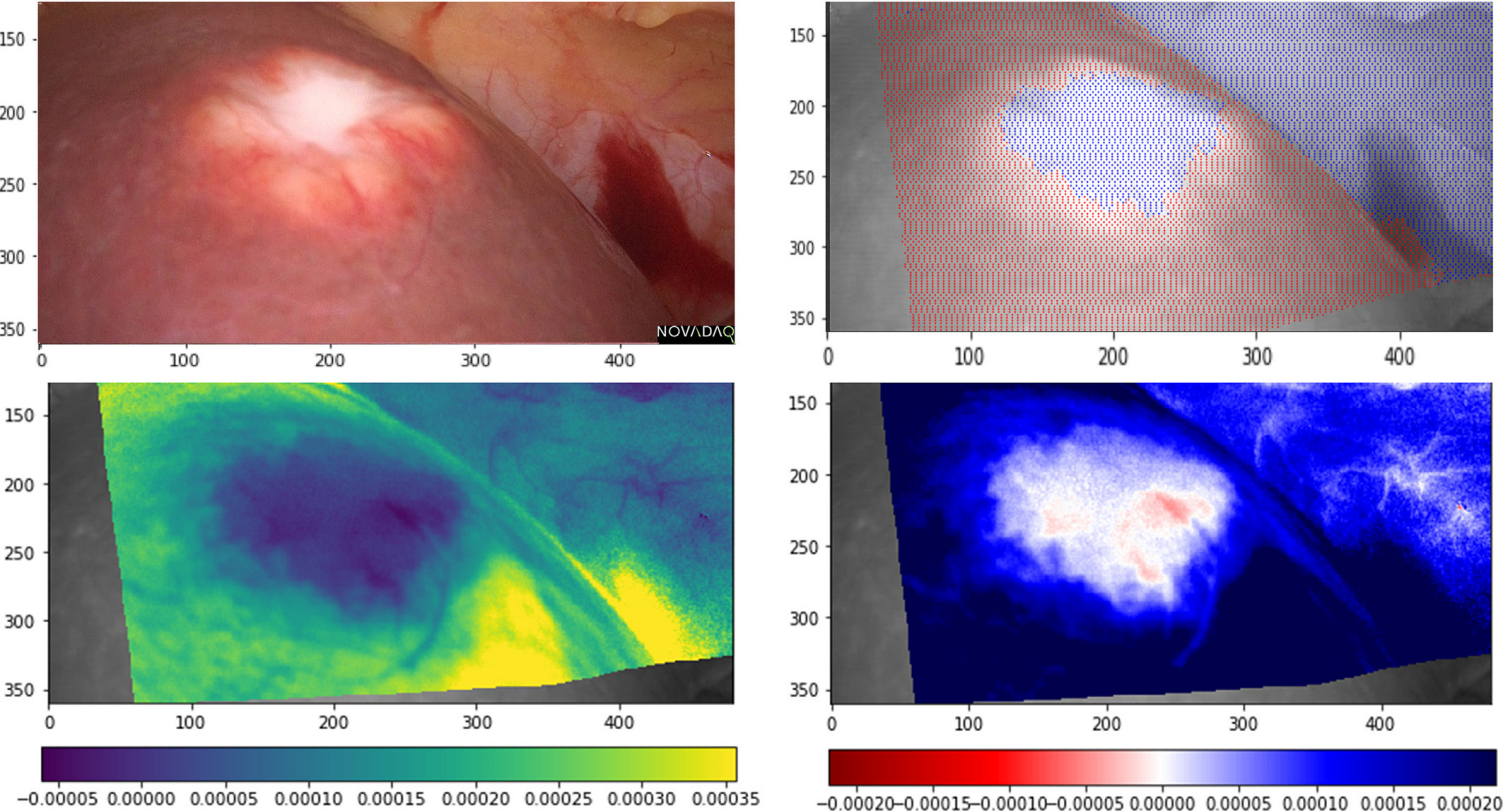
White light liver lesion photo with subsequent delineation using piecewise constant approximation of profiles followed by unsupervised clustering and outflow slope heatmaps. Slopes in [arbitrary units]/seconds.

Full field of view 2D perfusion mapping through full lesion image stabilization and pixel-by-pixel extraction was performed for 10 CRLMs (see Fig. 2 for examples). In addition, an ICG fluorescence angiogram of one benign liver lesion (a cyst) was also included displaying a lack of lesion differentiation versus surrounding healthy liver (see Fig. 2d). Indicative examples of the 2D representation, including using unsupervised clustering are shown in Fig. 3. All malignant lesions were successfully marginated including notably some poorly visible in white light viewing (n = 2).

**Fig. 4:**
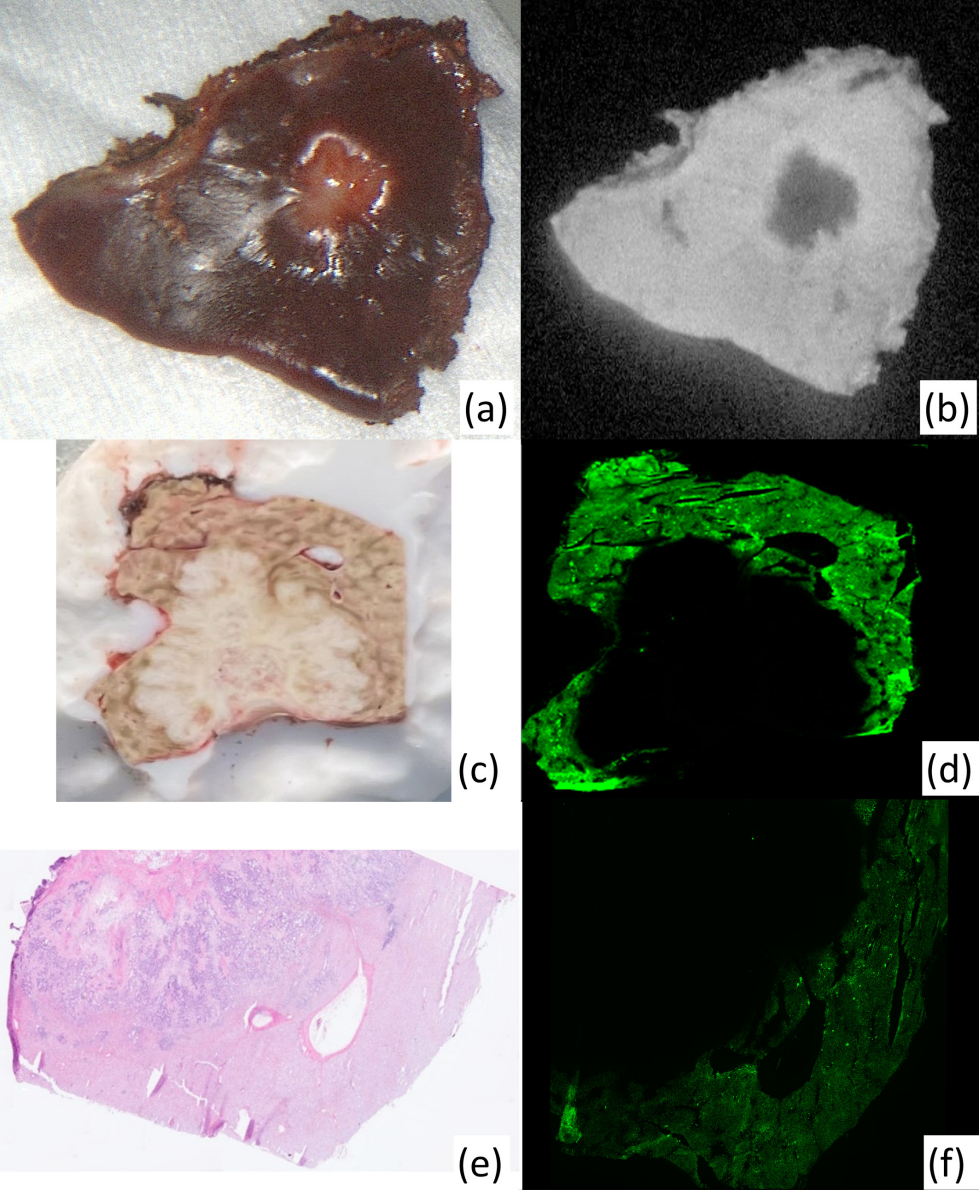
Macro and microscopic appearances of CRLMs and healthy liver tissue. (a) White light image of excised CRLM with surrounding healthy liver. (b) Black and white near infrared fluorescence image of lesion seen in (a) with early rim enhancement of CRLM demonstrated. (c) Frozen tissue sample comprising CRLM with surrounding healthy liver prior to cutting. (d) Microscopic appearances of sample seen in (c) with strong fluorescence signal seen in peripheral liver tissue only. (e) Microscopic appearances of CRLM with surrounding healthy liver tissue with Haematoxylin and Eosin staining. (f) Microscopic fluorescence imaging of unstained image (e) equivalent demonstrating the relative lack of ICG accumulation within the CRLM compared to the surrounding healthy liver tissue.

On microscopic interrogation, healthy liver parenchyma was statistically more fluorescent than the adjacent colorectal liver metastasis (p < 0.001) with mean (±std deviation) signal/area values of 507.43 (±160.82) and 2.46 (±9.56) for healthy liver tissue and CRLM respectively (see Fig. 4 for examples). Interestingly, samples treated with H&E staining and cover slipping had, on average, 6.5 times less fluorescence signal compared to identical fresh frozen unstained samples although importantly the distinctive fluorescence contrast differential persisted.

## Discussion

Evidence continues to accrue in favor of a minimally invasive, parenchymal preserving approach to CLRM resection. The inherent loss of tactile feedback in this mandates better intra-operative guidance [18,19]. While ICG is already used in the detection of liver lesions it remains limited in its usefulness with concerns expressed around dosing, timing of administration and potentially variation in camera systems influencing image interpretation and tumour specificity [10,20]. In this work we detail a methodology with the potential to address these issues and simplify clinical protocols. This approach, utilizing intraoperative administration with AI characterisation, could obviate the need for advance planning and permit prompt interrogation of lesions without the need for antecedent dosing and unlike intraoperative ultrasound does not require user interpretation. The method is also of potential use where liver lesions in need of characterising are encountered unexpectedly for the first-time during surgery for another indication (such as the colorectal surgeon encountering an indeterminate liver lesion during planned colon surgery).

Preceding experimental efforts had identified dosing at 0.05 mg/kg as the most optimal to permit a wide range of pixel intensity utilization (0–255 possible pixel intensity values in standard 8 bit black/white NIR imaging) without signal saturation which avoids the need for patient-by-patient dose and timing regimens as suggested in current guidelines [10,16]. As each patient provides their own control healthy reference, intra-patient comparisons of flow can be used to discriminate tissue type with inter-patient appearances, and indeed performance differences between imaging systems, of lesser importance. Within this study three different commercially available imaging systems were used at open, laparoscopic, and robotic surgery without the need for user interpretation of the resulting fluorescence signals. This counters concerns that some difficulties surrounding fluorescence interpretation are due to the differing fluorescence displays based on the imaging system and also work showing significant variations in performance between device manufacturers [10,21].

Despite its potential for medical advancement, AI translation from small scale research to clinical integration faces challenges. Deep Learning based methods in particular have been criticised for their lack of explainability meaning both the medical profession as well as the wider community are uncertain as to how AI methods can best be incorporated into healthcare [22]. One approach to address this is to incorporate a fundamental understanding of the processes under interrogation prior to the application of any ML. The biophysics approach described here incorporates a science-based understanding of underlying tissue-ICG interactions prior to the deployment of the ML classifier with tangible differences between tissue types demonstrable both mathematically and microscopically. Additionally, unlike current single point-in-time ICG interrogation, dynamic profiling in combination with such ML methodologies promises improved performance with increasing datasets as well as possible extension to other liver pathologies such as hepatocellular carcinoma (the fundamental hypothesis being that different tissue types have discriminant perfusion signatures).

While the microscopic fluorescence patterns within liver tissue have previously been reported at later time intervals (hours/days), here we add to prior work demonstrating the early (minutes) appearances [23]. The ROI perfusion profiles demonstrate clearly the rapid entry of ICG into the CRLM (see blue and green curves, Fig. 2a) followed by prompt washout with retention within the surrounding compressed (and thus functionally impaired) liver. Increased fluorescence within the rim surrounding the CRLM can be appreciated within minutes despite considerable ICG signal throughout the entire liver (see Fig. 4b). Furthermore, through objective quantification of tissue fluorescence signals we demonstrate the importance of tissue preparation methods on the fluorescence signal and the implication this may have on subsequent readings, with standard H&E preparation (the most commonly reported tissue preparation) causing a significant loss in the observable signal when compared with unstained tissue [24,25].

This work is limited by a relatively small patient cohort however given the underlying biophysics principles upon which the work is based, such findings will likely be consistent across larger sample sizes. Other limitations of the approach described here include the post hoc nature of the analysis as well as the limited depth of liver interrogation with only capsular and shallow subcapsular liver lesions readily visible with current NIR systems. With confidence of feasibility however we are moving towards real time methods of analysis (with automated whole screen tissue analysis) and next generation imaging systems allowing a greater depth of penetration (potentially permitting the creation of 3-dimensional tissue representation based on detected fluorescence signal) are already in progress. [[26], [27], [28]] Although whole screen/2D images delineating CRLM from surrounding healthy liver tissue can easily and reliably be generated with this methodology, further validation-optimisation with clinical implementation, including accuracy of margination determination, is ongoing. While some of the liver lesions will have undergone systemic treatment, all included patients were considered potential candidates for resection at the time of assessment and, in general, had healthy liver tissue without cirrhosis. As moderate/severe cirrhosis is known to alter liver ICG excretion, its presence may cause alterations in dynamic perfusion profiles and would likely need to be accounted for prior to algorithmic analysis in such patients (i.e. the addition of sample cirrhotic liver profiles to ML training sets thus permitting recognition of abnormal, but non-malignant tissue) [29].

In conclusion, ICG fluorescence angiograms in combination with computer vision and explainable ML methods can rapidly and accurately characterise capsular and subcapsular CRLMs on both a region-of-interest sample and full lesion basis.

## Funding sources

Work supported by Disruptive Technologies Innovation Fund, 10.13039/501100001588Enterprise Ireland, Ireland and 10.13039/100010477Intuitive Surgical, Sunnyvale California, USA. None of the listed authors and/or family members have any financial interests in the listed organisations.

## Ethics approval

All research was carried out with full Institutional Review Board (IRB) ethical approval (approval ref.: 1/378/2092) as part of a registered clinical trial: NCT 04220242.

## CRediT authorship contribution statement

Niall P Hardy: Conceptualization, Methodology, Formal analysis, Investigation, Data curation, Validation, Manuscript writing. Jonathan P Epperlein: Methodology, Formal analysis, Software. Jeffrey Dalli: Investigation, Resources, Data curation. William Robertson: Methodology, Investigation, Validation. Richard Liddy: Methodology, Investigation, Formal analysis, Validation. John Aird: Methodology, Investigation, Formal analysis, Validation. Niall Mulligan: Methodology, Investigation, Formal analysis, Validation. Peter M Neary: Conceptualisation, Methodology. Gerard P McEntee: Investigation, Resources. John B Conneely: Conceptualisation, Investigation, Resources. Ronan A Cahill: Conceptualization, Funding acquisition, Methodology, Formal analysis, Validation, Manuscript writing, Supervision.

## Declaration of competing interest

The authors declare the following financial interests/personal relationships which may be considered as potential competing interests: Professor Ronan A Cahill is named on a patent filed in relation to processes for visual determination of tissue biology, receives speaker fees from Stryker Corp and Ethicon/J&J, research funding from Intuitive Corp and Medtronic and holds research funding from the Irish Government (DTIF) in collaboration with IBM Research in Ireland and from EU Horizon 2020 in collaboration with Palliare. Dr Jeffrey Dalli and Dr Niall P Hardy are employed as researchers in the D.T.I.F. and Dr Jeffrey Dalli is recipient of the TESS scholarship (Malta). Dr Jonathan P Epperlein, Dr Peter M Neary, Dr John Aird, Dr Niall Mulligan, Mr. William Robertson, Dr Gerard P McEntee and Dr John B Conneely report no disclosures.
